# Cold climate and obesity in China: a cross-sectional ecological analysis

**DOI:** 10.3389/fpubh.2025.1722366

**Published:** 2026-01-12

**Authors:** Fuwei Yin, Yonghong Bao, Dahu Sun, Yang Zhang

**Affiliations:** 1 College of Physical Education, Hunan Normal University, Changsha, China; 2 Department of Physical Education, Central South University, Changsha, China; 3 Independent Person, Windermere, FL, United States

**Keywords:** adiponectin, climate, overweight, geographically weighted regression, sympathetic nervous system

## Abstract

**Background:**

Most research on obesity has focused on dietary, behavioral, and socioeconomic determinants. Environmental exposures such as climate, however, may also shape geographic variation in obesity, yet evidence from China remains scarce. Understanding whether cold exposure contributes to obesity distribution is important for broadening the framework of obesity prevention.

**Methods:**

We conducted an ecological analysis of 26 mainland Chinese provinces. Annual cold days (<4 °C) were averaged from 2000 to 2021. Overweight and obesity prevalence in 2019, derived from a national surveillance dataset of 15.8 million adults, were linked to climatic exposures. Associations were examined using ordinary least squares (OLS) regression and geographically weighted regression (GWR) to account for spatial heterogeneity. Sex differences were tested with pooled log–log models including interaction terms.

**Results:**

OLS residuals exhibited spatial autocorrelation, which was resolved by GWR. GWR revealed pronounced geographic variation: associations between cold days and overweight/obesity were strongest in transitional eastern provinces (Shanghai, Jiangsu, Zhejiang, Anhui; coefficients 0.40–0.48), whereas northern and western provinces showed relatively weaker or nonsignificant associations. In pooled models, the cold–overweight association was stronger in women than men (*p* = 0.009). For obesity, sex differences were not statistically significant (*p* = 0.33).

**Conclusion:**

Chronic cold exposure contributes to the spatial distribution of obesity in China, with the strongest effects in transitional eastern provinces. Climate should be recognized alongside socioeconomic and behavioral determinants in obesity epidemiology and considered in the design of regionally tailored prevention strategies.

## 1 Introduction

The prevalence of overweight and obesity has risen sharply over the past several decades, creating a major public health challenge worldwide. Recent forecasts suggest that if current trends continue, more than half of the global adult population will be classified as overweight or obese by 2050 (1). In China, the trajectory mirrors the global epidemic but is accelerating more rapidly. A nationwide survey of 15.8 million adults conducted in 2019 reported that more than one-third of Chinese adults were overweight and over 14 percent were obese under the World Health Organization (WHO) classification, with substantial burdens of complications such as fatty liver disease, prediabetes, dyslipidemia, and hypertension (2). These findings underscore the urgency of identifying not only behavioral but also environmental factors that may contribute to the growing prevalence of obesity.

Regional variation in obesity prevalence within China is well documented. Research on dietary and lifestyle patterns has highlighted the role of traditional wheat-based diets in the north compared with rice-based diets in the south, along with differences in physical activity and socioeconomic development (3). Urbanization has also played a significant role in shaping obesity patterns within China. Rapid expansion of urban environments has been linked to reduced physical activity, increased reliance on motorized transport, and greater access to energy-dense foods, all of which contribute to higher obesity risk (4). Recent analyses further show that higher provincial urbanization levels are associated with increased body mass index (BMI) and obesity prevalence among Chinese adults (5). Yet these behavioral and socioeconomic explanations do not fully account for the geographic clustering of obesity, raising the possibility that ecological exposures, including temperature, also play a role. Supporting this view, studies in both adults and younger populations consistently show a north–south gradient, with higher obesity prevalence in northern, higher-latitude provinces compared with southern regions (6). For example, a large-scale survey of more than 200,000 Chinese children and adolescents reported substantially higher obesity prevalence in northern provinces than in southern regions, even after accounting for age and sex (7), suggesting that latitude and associated climatic conditions may contribute to these disparities.

Experimental studies provide a biological rationale for considering temperature as a determinant of metabolic health. Cold exposure activates the renin–angiotensin system (RAS) and sympathetic nervous system (SNS), raising blood pressure and altering energy metabolism (8, 9). In a mouse model, cold suppresses adiponectin expression in white adipose tissue and lowers circulating adiponectin levels through sympathetic signaling (10). Because adiponectin regulates insulin sensitivity and thermogenesis, reduced activity may predispose to obesity. On this basis, the duration of cold exposure may provide a biologically meaningful indicator of environmentally relevant cold stress. Despite extensive research on cardiovascular outcomes (11), little is known about whether chronic cold exposure contributes to variation in obesity prevalence. Most population-level studies in China have focused on diet and lifestyle behaviors, leaving the potential influence of climate largely unexplored. Clarifying the contribution of temperature to regional variation in obesity prevalence is important for understanding the complex interplay between environment and metabolism and for anticipating how climate variability and population aging may interact with the obesity epidemic.

Recent spatial epidemiology research supports the importance of geographic and environmental determinants in shaping population-level obesity patterns (12). For example, a national spatial analysis in Türkiye found that multiple environmental factors—including elevation, precipitation, and slope—show spatially varying associations with adult obesity (13). This growing body of evidence reinforces the need to examine environmental exposures such as climate when interpreting regional obesity disparities.

Therefore, the present study aimed to examine whether chronic cold exposure is associated with the prevalence of overweight and obesity across Chinese provinces. If cold exposure is shown to be an important correlate of obesity prevalence, public health strategies may need to consider environmental as well as behavioral factors, informing prevention and intervention efforts in regions with prolonged cold seasons.

## 2 Methods

### 2.1 Study design

This ecological cross-sectional study examined the association between provincial-level prevalence of overweight and obesity and chronic cold exposure across mainland China. The unit of analysis was the provincial-level administrative region. The analysis relied exclusively on aggregated data from previously published sources, with no individual-level identifiers. Accordingly, institutional review board approval and informed consent were not required.

### 2.2 Data preparation

The outcomes were the provincial-level prevalence of overweight and obesity. These data were obtained from a large-scale cross-sectional survey published by Chen et al., which analyzed health check-up records from 15.8 million adults across 519 centers in 243 cities in 2019 (2). In this study, “overweight” and “obesity” follow the WHO adult BMI criteria, where overweight is defined as a BMI of 25.0–29.9 kg/m^2^ and obesity is defined as a BMI ≥ 30.0 kg/m^2^. Prevalence values reflect the proportion of adults within each province who met these WHO thresholds.

The exposure was the average annual number of days with a mean daily temperature below 4 °C. In this study, “cold days,” “<4 °C days,” and “chronic cold exposure” all refer to the same exposure metric. These terms are used interchangeably for stylistic variation but denote an identical province-level measure. The choice of the 4 °C threshold was guided by experimental evidence showing that temperatures at or below this level alter metabolic function via sympathetic activation, including suppression of serum adiponectin in a mouse model (10). Because adiponectin is a key mediator of insulin sensitivity and obesity risk, days meeting this criterion provide a biologically meaningful indicator of cold exposure. Accordingly, the annual count of <4 °C days served as the study’s primary predictor.

Temperature data were obtained from a homogenized daily meteorological dataset described by Argiriou et al., which provides continuous records of mean, minimum, and maximum temperatures from 366 national and regional stations across China since 1960 (14). For this analysis, we extracted mean daily temperature records from 100 national stations for the period 2000–2021 to align with the timeframe of available obesity data.

Because the outcome variables (expressed as a percentage) and the predictor (count of days) were on different scales, and because the prevalence data were positively skewed, we applied natural log transformations to all variables (15). This transformation stabilized variance and facilitated comparability between the predictor and outcome variables. It also allowed the regression coefficients to be interpreted as elasticities because both the predictor and outcome were log-transformed. In this context, an elasticity represents the percent change in overweight/obesity prevalence associated with a 1% change in the number of cold days. This log–log specification enables straightforward comparison of relative changes across provinces.

### 2.3 Ordinary least squares regression

All analyses were conducted at the provincial level, with no regional grouping or aggregation applied in any of the statistical models. We began by fitting an ordinary least squares (OLS) regression model to estimate the global association between log-transformed overweight/obesity prevalence and cold days. To evaluate whether the OLS specification adequately accounted for spatial dependence, we tested model residuals for spatial autocorrelation using Global Moran’s Index. Spatial relationships were defined using a k-nearest neighbors (KNN) approach with row standardization, which ensures that each province contributes equally to the test regardless of differences in neighbor count.

We selected KNN rather than an inverse distance weighting scheme because of the uneven size and spacing of Chinese provinces. For example, Tibet covers a vast geographic area but has relatively few neighboring provinces, while Jiangsu is much smaller and densely surrounded by other provinces. Under inverse distance, Tibet would appear only weakly connected to the rest of China, while Jiangsu would exert disproportionately strong influence. KNN avoids this imbalance by assigning each province the same number of neighbors, making it more appropriate for capturing local spatial relationships in this dataset.

The number of neighbors was set to *k* = 6, exceeding the minimum requirement of two observations per local regression (one predictor plus intercept) while maintaining a sufficiently local definition of neighborhood structure given the sample size. If Moran’s Index indicated no significant autocorrelation in the residuals, the OLS model was retained as the final specification. If significant clustering was detected, spatial modeling was pursued.

### 2.4 Geographically weighted regression

When residuals from the OLS model indicated spatial dependence, we applied geographically weighted regression (GWR) to evaluate spatial heterogeneity in the association between cold days and overweight/obesity prevalence. We used an adaptive nearest-neighbor bandwidth, which is recommended when spatial units vary substantially in size and density, as is the case for Chinese provinces. Adaptive bandwidths allow each local regression to draw from a comparable number of neighboring observations, improving stability in sparsely distributed regions while maintaining spatial detail in densely clustered areas (16). The optimal bandwidth was selected using the golden search procedure, which minimizes the corrected Akaike Information Criterion (AIC). Because the ArcGIS default (minimum of 20 neighbors) was not feasible for a provincial dataset of this size, we specified a custom range of 6 to 20 neighbors.

We applied a bi-square kernel weighting function, which gradually decreases the influence of more distant observations and assigns zero weight at the boundary of the adaptive neighborhood. The bi-square kernel is widely recommended for local spatial modeling because it provides a clear cutoff, reduces noise from distant units, and yields more localized and interpretable parameter estimates compared with Gaussian kernels when sample sizes are modest (17). This approach reduces the influence of distant provinces, provides sharper local resolution, and adapts flexibly to differences in regional density.

Following GWR estimation, residuals were again tested for spatial autocorrelation using Moran’s Index. If residual clustering persisted, the model was considered potentially misspecified. If residuals showed no significant autocorrelation, the GWR was deemed to adequately capture the spatial dependence structure.

Finally, to test whether the association between overweight/obesity prevalence and cold days differed between men and women, we also fit a pooled regression including a sex indicator and an interaction term between cold days and sex. The model specification was defined as Equation 1:


$$\begin{array}{l} \log(Y_{\text{is}})=\beta_{0}+\beta_{1}\log(\text{ColdDay}s_{i})+\beta_{2}\text{Femal}e_{s}\\ +\beta_{3}[\log(\text{ColdDay}s_{i})\times \text{Femal}e_{s}] \end{array}$$ (1)

where $Y_{\text{is}}$ is the log-transformed prevalence of overweight/obesity for sex *s* in province *i*. The coefficient *β*_1_ estimates the elasticity for males, while *β*_2_ + *β*_3_ estimates the elasticity for females. The interaction term *β*_3_ provides a direct statistical test of whether women’s cold–overweight/obesity elasticity differs significantly from men’s. This pooled specification complements the spatial models by offering a global test of sex differences, whereas GWR highlights spatial heterogeneity across provinces.

**Figure 1:**
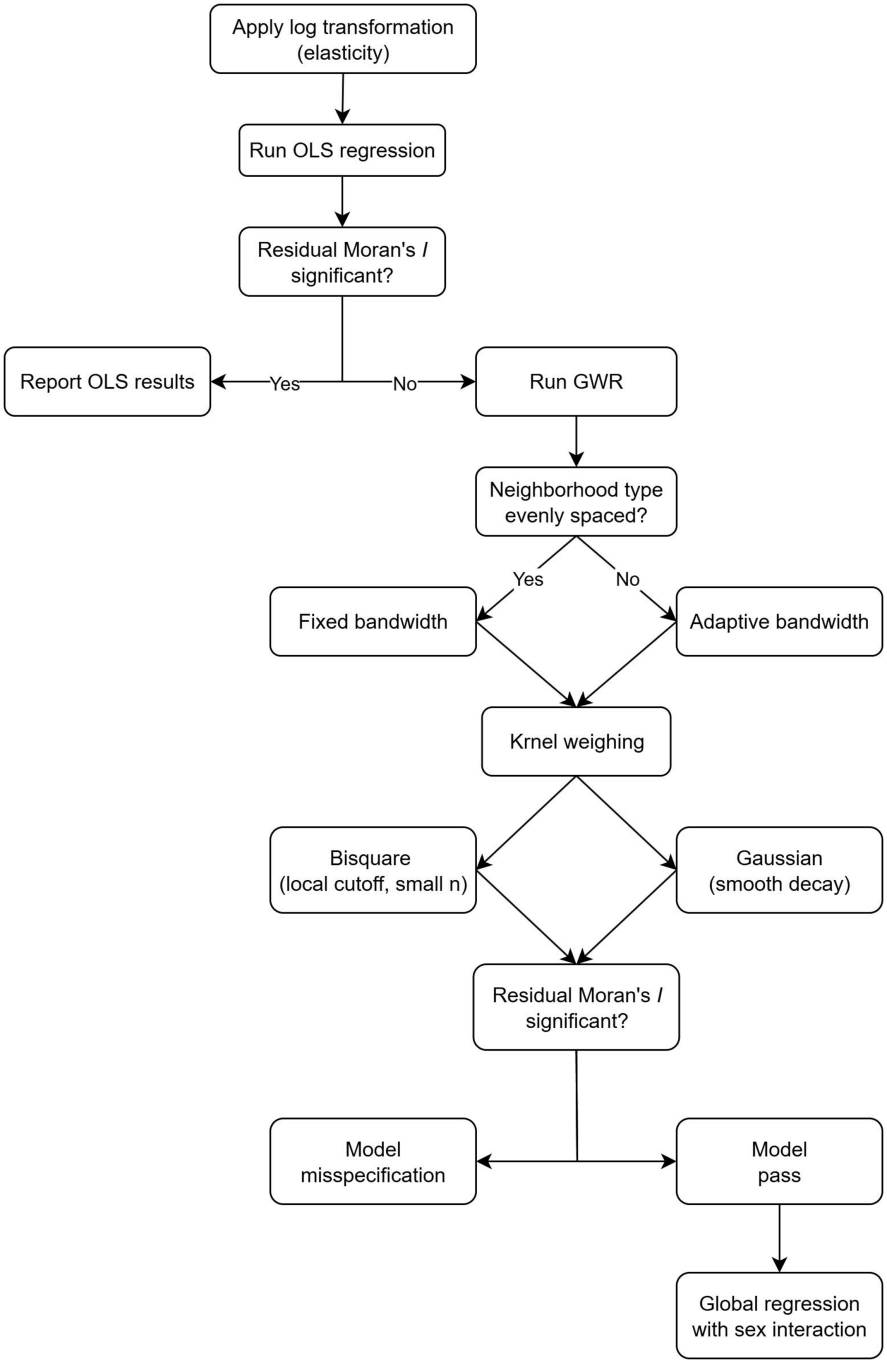
Flowchart of analytic approach.

The decision process of the analytic approach is summarized in Figure 1, which provides a visual overview of the steps linking the OLS model, spatial autocorrelation testing, and the application of GWR. Statistics were conducted using ArcGIS Pro (version 3.1.5) and RStudio. A two-sided *p*-value less than 0.05 was considered statistically significant. The dataset supporting the findings of this study is available in Figshare (DOI: 10.6084/m9.figshare.30260389).

## 3 Results

**Figure 2:**
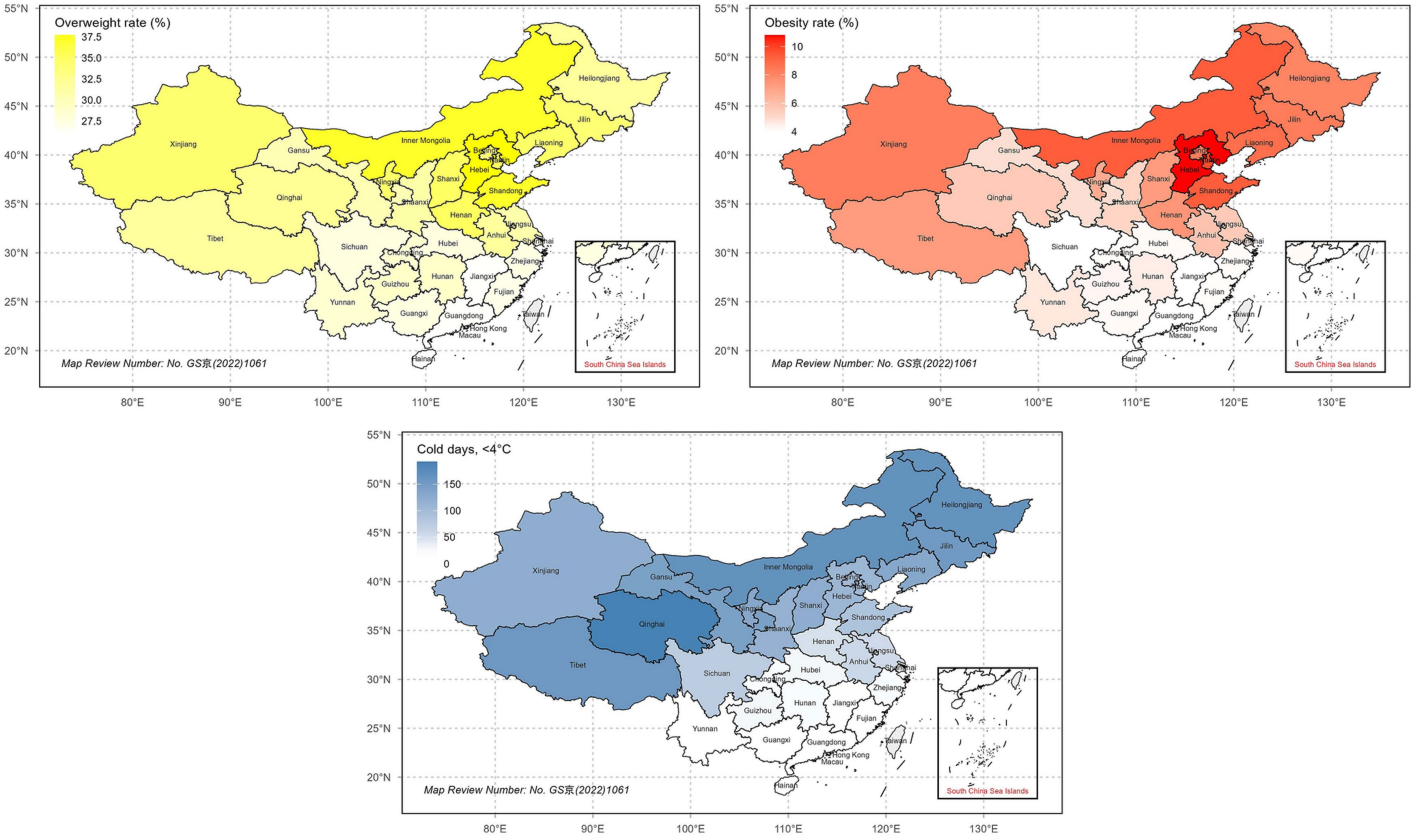
Provincial-level maps of overweight prevalence, obesity prevalence, and number of days with mean daily temperature below 4 °C across mainland China. Provinces without prevalence data (Taiwan, Hong Kong, and Macau) are shaded in gray.

Descriptive maps of overweight prevalence, obesity prevalence, and cold days are presented in Figure 2. The dataset comprised 31 administrative regions, with Hong Kong, Macau, and Taiwan excluded because obesity prevalence data were not available. Overweight prevalence ranged from approximately 26% in southern provinces to more than 40% in several northern provinces. Obesity prevalence ranged from about 4–5% in the south to more than 12% in parts of the north and east. The number of days with a mean daily temperature below 4 °C displayed a marked geographic gradient, with fewer than 2 days per year in far southern provinces, compared with more than 150 days in Inner Mongolia and Heilongjiang. Provinces with negligible cold exposure (<2 cold days per year), including Fujian, Guangdong, Guangxi, Hainan, and Yunnan, were excluded from subsequent analyses because they exhibit no meaningful variation in the exposure. Cold-induced metabolic pathways require sustained low-temperature exposure and cannot plausibly operate under such climatic conditions; including these units would introduce leverage-driven distortion due to structural zero exposure values rather than improve causal estimation. Moreover, applying log-based transformations to near-zero exposure values would further distort the exposure distribution for the entire dataset, potentially yielding spurious coefficients unrelated to meaningful climatic variation. Accordingly, their exclusion prevents exposure misclassification rather than introducing selection bias. The final analytic dataset consisted of 26 spatial units.

As shown in Table 1, OLS regression of overweight and obesity prevalence on cold days showed significant positive spatial autocorrelation in model residuals (all *p* < 0.001), indicating that the global specification did not adequately account for spatial dependence. In contrast, GWR successfully resolved spatial clustering, with no significant residual autocorrelation detected (all *p* > 0.25) (Table 1). Consistent with this improvement, GWR also demonstrated better overall model performance relative to OLS, as indicated by lower AIC_c_ values and higher R^2^ values across all six outcome specifications (Table 2). These results confirm that allowing coefficients to vary locally improved model fit and resolved the spatial dependence present in the OLS models. Therefore, subsequent analyses focused on GWR.

**Table 1:** Spatial autocorrelation of OLS/GWR residuals for overweight and obesity prevalence across 26 provinces in China.

Dependent variable	OLS		GWR	
	Moran’s index	*p*-value	Moran’s index	*p*-value
Overweight prevalence (male)	0.300	0.0001	−0.015	0.775
Overweight prevalence (female)	0.283	0.0003	−0.612	0.806
Overweight prevalence (overall)	0.328	<0.0001	−0.041	0.993
Obesity prevalence (male)	0.608	<0.0001	0.062	0.252
Obesity prevalence (female)	0.302	0.0001	0.005	0.616
Obesity prevalence (overall)	0.526	<0.0001	0.039	0.377

**Table 2:** Global model performance for OLS and GWR models.

Outcome and sex	OLS AIC_c_	GWR AIC_c_	ΔAICc (OLS–GWR)	OLS R^2^	GWR R^2^
Overweight—male	−64.0	−71.2	7.2	0.417	0.694
Overweight—female	−40.4	−46.6	6.2	0.590	0.777
Overweight—overall	−56.9	−64.3	7.4	0.530	0.755
Obesity—male	6.8	−12.7	19.5	0.437	0.816
Obesity—female	8.2	1.0	7.2	0.565	0.772
Obesity—overall	4.6	−9.6	14.2	0.509	0.804

AIC_c_ corrected Akaike Information Criterion. Lower values indicate better model fit. ΔAIC_c_ > 10 is generally interpreted as strong evidence for superior model performance.

**Figure 3:**
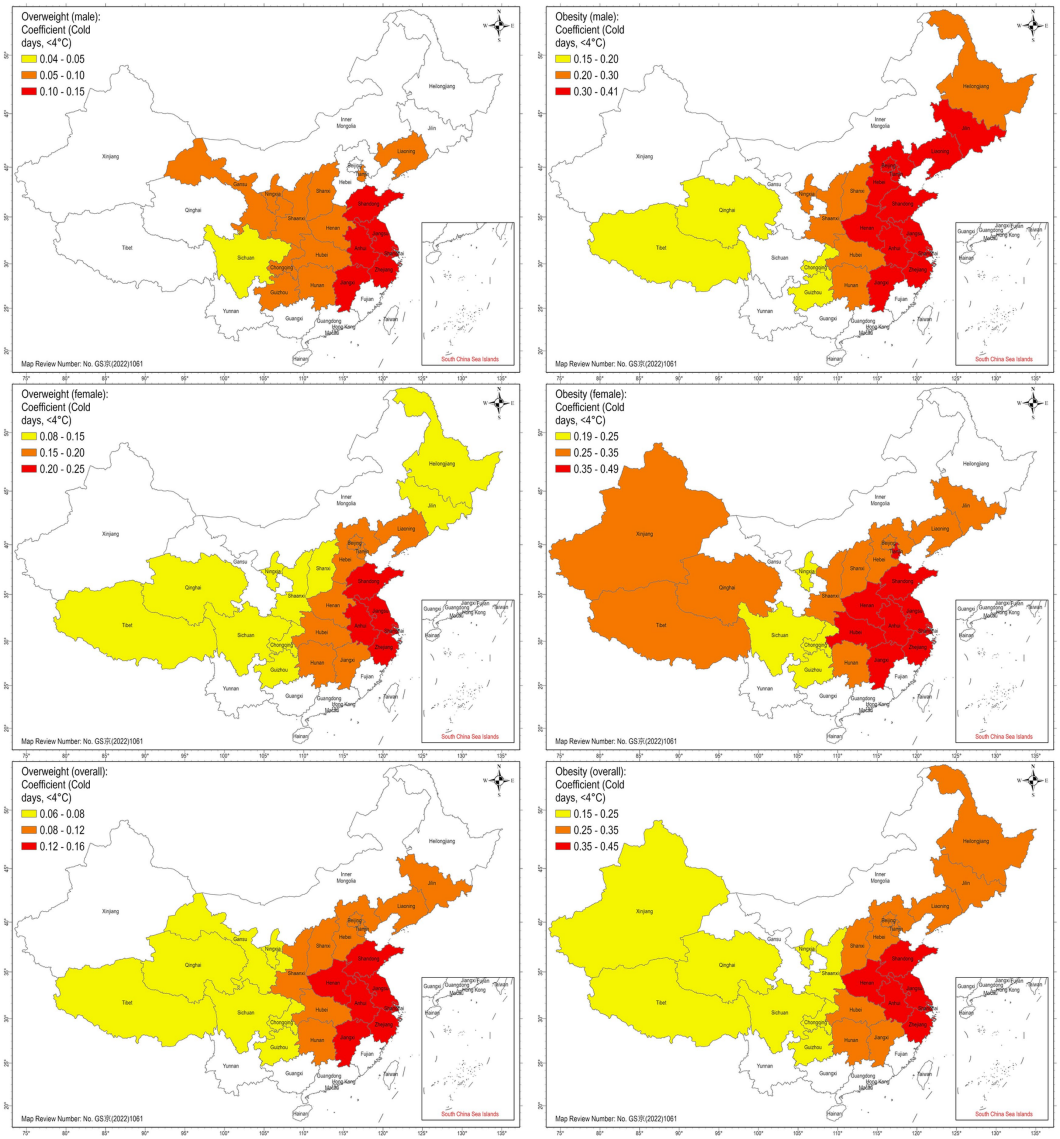
Geographically weighted regression coefficients for the association between cold days (<4 °C) and overweight/obesity prevalence. Provinces with nonsignificant coefficients or without data are shown in white.

GWR results are presented in Table 3 and Figure 3. Each local GWR model follows the same log–log specification as the global model, but the coefficients are estimated separately for each province using an adaptive nearest-neighbor bandwidth, resulting in spatially varying parameter estimates. Results indicate pronounced regional heterogeneity in the association between cold days and overweight/obesity prevalence. The strongest associations were observed in transitional eastern and coastal provinces, including Shanghai, Jiangsu, Zhejiang, and Anhui, where local coefficients for overall obesity ranged from 0.40 to 0.48. For illustration, a coefficient of 0.40 in Zhejiang implies that a 1% increase in the number of cold days is associated with an approximate 0.40% increase in obesity prevalence in that province, whereas a coefficient near 0.15 in Sichuan corresponds to a much weaker relationship. Of note, these examples are intended solely to clarify the interpretation of elasticities; given the ecological and cross-sectional nature of the data, the coefficients should be viewed as descriptive indicators rather than predictive or causal effects. In these eastern provinces, the corresponding local t-values exceeded the GWR-adjusted critical value (*t* = 2.491), indicating statistically meaningful local relationships. In contrast, provinces in the far north and west, despite experiencing the greatest number of cold days and having high overall obesity prevalence, showed local *t*-values below the adjusted threshold, suggesting weak or nonsignificant associations.

**Table 3:** GWR estimates for the association between cold days (<4 °C) and overweight/obesity prevalence across 26 provinces in China.

Province	Overweight (male)			Overweight (female)			Overweight (overall)			Obesity (male)			Obesity (female)			Obesity (overall)		
	*β*	*t*-value*	*R* ^2^	*β*	*t*-value*	*R* ^2^	*β*	*t*-value*	*R* ^2^	*β*	*t*-value*	*R* ^2^	*β*	*t*-value*	*R* ^2^	*β*	*t*-value*	*R* ^2^
Beijing	0.061	2.296	0.385	0.159	3.703	0.502	0.099	3.247	0.457	0.303	3.689	0.575	0.315	2.945	0.544	0.311	3.558	0.567
Tianjin	0.073	2.962	0.486	0.174	4.422	0.566	0.111	3.973	0.537	0.338	4.479	0.616	0.355	3.610	0.585	0.347	4.323	0.609
Hebei	0.066	2.454	0.392	0.164	3.803	0.509	0.103	3.375	0.465	0.310	3.758	0.579	0.329	3.065	0.554	0.320	3.655	0.574
Shanxi	0.069	2.560	0.403	0.147	3.413	0.530	0.097	3.158	0.477	0.273	3.298	0.624	0.309	2.863	0.624	0.289	3.287	0.628
Inner Mongolia	0.023	0.736	0.263	0.105	2.048	0.413	0.056	1.543	0.340	0.198	2.026	0.574	0.185	1.450	0.521	0.196	1.888	0.554
Liaoning	0.063	2.965	0.565	0.161	4.765	0.640	0.100	4.156	0.606	0.341	5.243	0.706	0.335	3.959	0.650	0.339	4.904	0.687
Jilin	0.053	2.404	0.509	0.149	4.229	0.609	0.090	3.576	0.559	0.327	4.825	0.705	0.312	3.531	0.632	0.320	4.455	0.679
Heilongjiang	0.024	0.853	0.327	0.115	2.576	0.426	0.060	1.896	0.364	0.273	3.186	0.575	0.232	2.076	0.458	0.257	2.825	0.528
Shanghai	0.114	5.800	0.822	0.221	7.008	0.845	0.150	6.673	0.842	0.399	6.580	0.830	0.478	6.064	0.857	0.423	6.581	0.848
Jiangsu	0.115	5.881	0.803	0.224	7.148	0.823	0.151	6.795	0.819	0.404	6.730	0.813	0.481	6.158	0.836	0.428	6.724	0.827
Zhejiang	0.111	5.676	0.808	0.214	6.800	0.839	0.145	6.480	0.836	0.377	6.239	0.820	0.465	5.909	0.859	0.403	6.289	0.845
Anhui	0.108	5.910	0.785	0.210	7.150	0.817	0.142	6.795	0.811	0.369	6.556	0.803	0.453	6.181	0.838	0.395	6.608	0.825
Jiangxi	0.100	4.986	0.749	0.192	5.953	0.800	0.130	5.658	0.795	0.316	5.115	0.766	0.408	5.071	0.831	0.343	5.218	0.807
Shandong	0.103	4.757	0.689	0.212	6.116	0.712	0.142	5.744	0.704	0.409	6.155	0.726	0.454	5.250	0.720	0.425	6.031	0.728
Henan	0.096	5.183	0.689	0.189	6.395	0.740	0.127	6.039	0.724	0.329	5.794	0.739	0.404	5.468	0.776	0.353	5.858	0.760
Hubei	0.090	4.872	0.699	0.172	5.828	0.758	0.117	5.563	0.747	0.286	5.036	0.736	0.362	4.910	0.794	0.307	5.104	0.769
Hunan	0.081	4.010	0.651	0.158	4.872	0.732	0.106	4.585	0.718	0.240	3.854	0.672	0.326	4.028	0.769	0.262	3.976	0.725
Chongqing	0.060	2.912	0.519	0.114	3.454	0.608	0.078	3.308	0.583	0.174	2.750	0.545	0.232	2.815	0.626	0.188	2.786	0.575
Sichuan	0.050	2.648	0.458	0.083	2.784	0.508	0.062	2.884	0.502	0.140	2.429	0.450	0.197	2.626	0.514	0.154	2.520	0.456
Guizhou	0.055	2.855	0.498	0.108	3.484	0.610	0.073	3.284	0.576	0.152	2.549	0.509	0.223	2.881	0.629	0.168	2.665	0.551
Tibet	0.045	2.081	0.353	0.098	2.845	0.522	0.065	2.639	0.478	0.170	2.574	0.453	0.335	3.889	0.542	0.231	3.281	0.522
Shaanxi	0.071	3.117	0.563	0.127	3.473	0.625	0.090	3.457	0.601	0.237	3.365	0.674	0.264	2.882	0.671	0.245	3.287	0.677
Gansu	0.059	2.530	0.508	0.082	2.194	0.516	0.067	2.540	0.520	0.170	2.384	0.593	0.207	2.232	0.505	0.181	2.392	0.551
Qinghai	0.051	2.352	0.432	0.089	2.552	0.494	0.066	2.635	0.479	0.170	2.533	0.482	0.277	3.163	0.535	0.208	2.910	0.504
Ningxia	0.063	2.932	0.558	0.102	2.991	0.611	0.076	3.131	0.589	0.200	3.049	0.696	0.219	2.562	0.651	0.206	2.956	0.681
Xinjiang	0.048	1.969	0.453	0.092	2.358	0.473	0.065	2.339	0.491	0.164	2.181	0.532	0.305	3.116	0.447	0.217	2.711	0.482

Local statistical significance is assessed by comparing the local *t*-values to the GWR-adjusted critical *t*-value 2.491 (*α* = 0.05). *p*-values are not reported because they are not recommended for local GWR coefficients due to multiple testing concerns. The optimal adaptive bandwidth selected for the GWR model included 19 nearest neighbors, meaning each local regression was estimated using the 19 closest provinces.

In pooled models, the overweight–cold association was significantly stronger among women than men (*β*_3_ = 0.081; 95% CI, 0.021–0.141; *p* = 0.009). By contrast, for obesity prevalence, the sex difference in slopes was not significant (*β*_3_ = 0.088; 95% CI, −0.092 to 0.267; *p* = 0.33). Thus, evidence of sex differences was observed for overweight but not for obesity.

## 4 Discussion

By linking a nationally representative dataset of 15.8 million adults with two decades of provincial climate records, this study provides the first ecological evidence at a national scale that chronic cold exposure is associated with obesity prevalence in China. We found that chronic cold exposure, operationalized as the annual number of days with a mean temperature below 4 °C, was associated with overweight and obesity prevalence, although with substantial geographic heterogeneity. These findings suggest that cold exposure may help explain the spatial distribution of obesity in China and may complement explanations rooted in socioeconomic and behavioral factors.

The observed associations are supported by several converging biological mechanisms. Cold exposure activates the RAS in the brain, increasing angiotensinogen mRNA expression and enhancing SNS outflow (18, 19). Centrally generated angiotensin II acts on circumventricular organs, including the subfornical organ and hypothalamic nuclei, to stimulate sympathetic efferents. These pathways then alter metabolic and vascular regulation. Importantly, such effects do not require uniform systemic SNS activation. Even selective activation of angiotensin II–sensitive brain regions can generate localized sympathoexcitation that influences metabolic hormones (18). In practical terms, this means that specific brain circuits can reduce adiponectin levels without raising sympathetic tone throughout the entire body. Experimental work supports this mechanism: sympathetic activation suppresses adiponectin mRNA expression in adipose tissue and lowers circulating adiponectin levels (10). Adiponectin regulates thermogenesis, energy expenditure, and insulin sensitivity; therefore, its suppression promotes positive energy balance and weight gain (20–23). In animal models, adiponectin is required for maintaining body temperature in cold environments (24), underscoring its role as a mediator of thermal stress. Acute cold exposure reduces serum adiponectin concentrations through SNS activation (10), reinforcing the plausibility of a temperature–adiponectin link. Low adiponectin is also consistently associated with obesity, insulin resistance, and cardiovascular disease (25, 26), suggesting that a climate–adiponectin connection may be an underrecognized contributor to geographic obesity variation.

Cold weather also influences health through behavioral pathways. Colder conditions reduce outdoor physical activity and increase sedentary time. Seasonal dietary changes contribute as well: winter months are associated with higher consumption of calorie-dense foods (27). Although once adaptive, this pattern may now promote weight gain in modern urban settings with abundant food availability. These shifts in diet and activity favor a positive energy balance during colder months. Such mechanisms may be especially relevant in highly urbanized provinces, where sedentary lifestyles and energy-dense diets magnify the effects of environmental exposures. These interacting pathways offer a possible explanation for the regional heterogeneity observed in our analysis. In transitional eastern provinces, where winters are moderately cold but not extreme, climatic exposure appears sufficient to activate both biological and behavioral mechanisms. Rapid urbanization, sedentary lifestyles, and high-calorie diets likely amplify these effects.

A historical lack of winter heating in many of these provinces may also play a role. Unlike the far colder northern provinces—with long-standing centralized heating infrastructure—populations in Shanghai, Jiangsu, Zhejiang, and Anhui traditionally endured winter cold with limited indoor insulation. This insufficient heating may increase reliance on physiological thermoregulation, including brain angiotensin II–mediated sympathetic activation. As a result, individuals in these provinces could theoretically experience more pronounced cold-related reductions in adiponectin compared with northern residents whose indoor heating blunts exposure. This context may help explain why estimated coefficients were strongest in transitional provinces, where moderate but unbuffered cold intersects with inadequate adaptation. In such regions, even modest climatic stress may initiate SNS pathways that intensify adiponectin suppression and energy imbalance.

By contrast, in the far north and west, obesity prevalence is high but marginal effects of additional cold days are weak. This pattern may reflect ceiling effects or long-term adaptation. Populations in these regions have adapted physiologically and culturally to harsh winters. They have better housing insulation, established heating infrastructure, and dietary practices suited for extreme cold. In southern provinces, where cold exposure is minimal, overweight and obesity prevalence remains among the lowest nationwide. These patterns underscore that climate interacts with socioeconomic context: extreme cold provinces may have adaptations that blunt incremental climatic effects, while transitional eastern provinces experience moderate cold combined with limited heating and urbanized lifestyles that heighten vulnerability.

Beyond geographic heterogeneity, the association between chronic cold exposure and overweight appeared stronger in women than in men. The mechanisms underlying this sex difference remain uncertain, and existing evidence is mixed. Rodent studies suggest that estrogen may attenuate cold-induced metabolic responses, including adiponectin upregulation and beige fat recruitment (28). Under estrogen-deficient conditions, thermogenic responses to cold are often enhanced. Whether these pathways translate to humans is unclear, and human data on estrogen and cold-induced adiponectin dynamics are limited.

Behavioral factors may offer more plausible explanations. Women often exhibit larger seasonal reductions in outdoor physical activity, greater sensitivity to cold discomfort, and different patterns of clothing or heating use (29, 30). These factors may magnify the metabolic consequences of prolonged cold exposure. Given the ecological design, this sex difference should be interpreted cautiously. Future studies with individual-level hormone, adiponectin, and thermogenic measurements are needed to clarify whether biological or behavioral mechanisms explain the observed pattern.

The findings have implications for public health and policy. Environmental exposures such as climate should be considered alongside socioeconomic, dietary, and behavioral determinants of obesity. In transitional eastern provinces, should causal pathways be confirmed, interventions may include promoting wintertime physical activity and targeted health campaigns. Practical options include subsidizing home-based exercise equipment, developing digital programs that encourage winter activity, and providing indoor exercise spaces in schools and workplaces. Precision nutrition approaches that account for winter reductions in activity may help counter positive energy balance. In northern provinces, where cold is constant and marginal effects are small, strategies should prioritize lifestyle factors such as diet quality.

Climate change may also alter the geographic distribution of obesity risk. Shorter, milder winters could reduce climate-related risk in northern provinces but may shift vulnerabilities in transitional regions. Anticipating such changes will be important for designing adaptive strategies in national obesity prevention efforts.

Several limitations should be considered. First, given the ecological and cross-sectional design, no causal inferences can be drawn, and all associations identified here should be interpreted as hypothesis-generating rather than causal. The unit of analysis was the province; therefore, ecological inference to individuals is not possible, and temporality cannot be established. Second, adiponectin was not measured in this study. Thus, the proposed cold–adiponectin–obesity pathway should be interpreted as a theoretical framework supported by prior experimental literature (10, 23) but not empirically evaluated in our analysis. Accordingly, any discussion of adiponectin is intended to contextualize possible biological mechanisms rather than to imply mechanistic confirmation. Third, measurement error is possible because provincial averages of cold days may not fully capture microclimate variation, elevation differences, or urban heat island effects, especially in large metropolitan areas. Fourth, direct indicators of indoor temperature, heating infrastructure, and household energy use were unavailable across all provinces and years, which limits characterization of actual thermal exposure. Fifth, additional spatial covariates such as urbanization, GDP per capita, and healthcare access may influence provincial obesity patterns. Some of these indicators are available; however, integrating them into the current analysis would shift the focus toward building a multivariable predictive model rather than examining historical ecological associations between chronic cold exposure and obesity prevalence. Moreover, the cross-sectional ecological design would still preclude causal inference even with statistical adjustments. Nonetheless, the absence of these controls introduces the possibility of residual confounding and should be acknowledged. Sixth, while the ecological associations identified here are informative at the population level, they may not translate directly to individual risk without validation in longitudinal or multilevel cohort studies. Finally, replication in other national or subnational datasets would be valuable to assess generalizability beyond China, and simulation or sensitivity modeling of adiponectin-related pathways may further strengthen biological plausibility in subsequent analyses.

In conclusion, this study provides national ecological evidence that chronic cold exposure is associated with provincial variation in obesity prevalence. Our findings underscore that climate may play a contextual role alongside socioeconomic and behavioral factors. Recognizing climate as part of the obesity framework is important for developing regionally tailored prevention strategies, especially in transitional provinces that lack widespread winter heating. As climate change alters winter severity, integrating environmental exposures into surveillance and prevention planning will become increasingly essential.

## Data Availability

Publicly available datasets were analyzed in this study. This data can be found at: The dataset supporting the findings of this study is available in Figshare (DOI: 10.6084/m9.figshare.30260389).
